# A Full MALDI-Based Approach to Detect Plasmid-Encoded KPC-Producing *Klebsiella pneumoniae*

**DOI:** 10.3389/fmicb.2018.02854

**Published:** 2018-11-23

**Authors:** Miriam Cordovana, Markus Kostrzewa, Jörg Glandorf, Michael Bienia, Simone Ambretti, Arthur B. Pranada

**Affiliations:** ^1^Laboratory of Bacteriology, Operative Unit of Microbiology, University Hospital of Bologna Policlinico Sant’Orsola-Malpighi, Bologna, Italy; ^2^Bruker Daltonik GmbH, Bremen, Germany; ^3^Department of Medical Microbiology, MVZ Dr. Eberhard & Partner Dortmund, Dortmund, Germany

**Keywords:** MALDI-TOF MS, KPC, *Klebsiella pneumoniae*, carbapenemase, multidrug resistance, KPC-related peak, pKpQIL plasmid

## Abstract

KPC-producing *Klebsiella pneumoniae* represents a severe public health concern worldwide. The rapid detection of these isolates is of fundamental importance for the adoption of proper antibiotic treatment and infection control measures, and new applications of MALDI-TOF MS technology fit this purpose. In this study, we present a full MALDI-based approach to detect plasmid-encoded KPC-producing strains, accomplished by the automated detection of a KPC-specific peak (at 11,109 m/z) by a specific algorithm integrated into the MALDI Biotyper system (Bruker Daltonik), and the confirmation of carbapenemase activity by STAR-Carba imipenem hydrolysis assay. A total of 6209 *K. pneumoniae* isolates from Italy and Germany were investigated for the presence of the KPC-related peak, and a subset of them (*n* = 243) underwent confirmation of carbapenemase activity by STAR-Carba assay. The novel approach was further applied directly to positive blood culture bottles (*n* = 204), using the bacterial pellet obtained with Sepsityper kit (Bruker Daltonik). The novel approach enabled a reliable and very fast detection of KPC-producing *K. pneumoniae* strains, from colonies as well as directly from positive blood cultures. The automated peak detection enabled the instant detection of KPC-producing *K. pneumoniae* during the routine identification process, with excellent specificity (100%) and a good sensitivity (85.1%). The sensitivity is likely mainly related to the prevalence of the specific plasmid harboring clones among all the KPC-producing circulating strains. STAR-Carba carbapenemase confirmation showed 100% sensitivity and specificity, both from colonies and from positive blood cultures.

## Introduction

Carbapenem-resistant *Enterobacteriaceae* (CRE) have recently emerged as a class of bacterial pathogens that threaten the effectiveness of last resort treatment, posing a serious threat to global public health (Tzouvelekis et al., 2012). Even though resistance to carbapenems may involve several combined mechanisms (Nordmann et al., 2012a), from the public health point of view attention is focused on the isolates that produce specific carbapenem-hydrolyzing β-lactamases (carbapenemases). Such resistance determinants can be transferred between bacteria of the same or of different species by mobile genetic elements (Kumarasamy et al., 2010; Nordmann et al., 2012b). The carbapenemase transmission often is linked to other non-β-lactam resistance determinants, leading to the rise and the rapid dissemination of multi-drug or pan-drug resistant (MDR, PDR) organisms (Kumarasamy et al., 2010).

Carbapenemase-producing *Enterobacteriaceae* (CPE) have become important causes of hospital acquired infections, associated with a significantly increased mortality, especially in critical wards (Vincent et al., 2009). In Europe, the highest prevalences have been reported from Greece, with 61.9% of carbapenem-resistant *K. pneumoniae* among invasive isolates, Italy with 33.5%, and from Romania (24.7%) (EARS-Net report 2016).

CPE show an epidemiology according to the enzyme type, with different prevalence in the different geographic areas. In Europe, KPC-producing *K. pneumoniae* is endemic in Italy and Greece, while in other countries class D (OXA-48 family) and class B (MβL - Metallo-β-Lactamases) carbapenemases are reported with overall lower prevalence. Nevertheless, the *Klebsiella pneumoniae* Carbapenemase (KPC) family has the most extensive global distribution of all carbapenemases associated with *Enterobacterales* (van Duin and Doi, 2017), and are the most clinically significant (Munoz-Price et al., 2013). KPCs are found in many Gram-negative species, including both *Enterobacterales* and non-fermenters, but *K. pneumoniae* is the most largely predominant species.

Actually, 20 variants of the *bla*KPC gene have been described, and the variants KPC-2 and KPC-3 are the most common (Doumith et al., 2017). The *bla*_KPC_ genes have been identified in a variety of plasmids (Chen et al., 2014), and reported in more than 100 different Sequence Types (ST) of *K. pneumoniae*. The transmission of *bla*_KPC_ genes can be mediated by different molecular mechanisms, from mobility of small genetic elements (i.e., Tn*4401* transposon) to horizontal transfer of plasmids, and via clonal spread (Munoz-Price and Quinn, 2009; Partridge, 2014). Nevertheless, the global diffusion of plasmid-borne *bla*_KPC_ has been linked to the clonal dissemination worldwide of a major multilocus sequence type (MLST or ST), namely ST258, and its related variants (Cuzon et al., 2010; Munoz-Price et al., 2013). KPC-Kp are considered endemic in several countries, including the North-eastern United States, Argentina, Brazil, Puerto Rico, Colombia, China, Israel, and, in Europe, Italy and Greece (Munoz-Price et al., 2013; Nordmann and Poirel, 2014). As treatment options are very limited and because of the epidemiological impact, rapid methods to detect CPE, and differentiate them from other CRE are desired. Several techniques have been developed, relying on different molecular or phenotypical principles. Although proven to be effective for the detection of KPC-producing isolates, overall these methods are either expensive and limited in the targets included, or slow with a time to report up to 24 h, and lack in sensitivity and/or specificity (Osei Sekyere et al., 2015; Bialvaei et al., 2016; Lutgring and Limbago, 2016; Rood and Li, 2017; Tamma et al., 2017).

Recently, it has been shown that the intrinsic speed of MALDI-TOF MS technology could be deployed and successfully applied to a prompt detection of carbapenemase-producing strains. First, it was proven that the hydrolytic activity of carbapenemases can be detected by a functional assay that relies on the evaluation of the mass spectra of the carbapenem molecule after a short incubation with the bacterial strains (Burckhardt and Zimmermann, 2011; Sparbier et al., 2012; Hrabák et al., 2013; Johansson et al., 2014; Rapp et al., 2018). This is even possible directly from positive blood cultures (Jung et al., 2014; Hoyos-Mallecot et al., 2014; Sakarikou et al., 2017).

Further, Lau et al. (2014) discovered a peak in MALDI-TOF mass spectra of KPC-producing *K. pneumoniae* related to a pKpQIL plasmid carrying *bla*_KPC_. This specific peak at 11,109 m/z is clearly detectable in bacterial MALDI-TOF mass spectra. Different approaches to seek for this peak have been used and evaluated (Lau et al., 2014; Gaibani et al., 2016; Youn et al., 2016). Until recently, the detection of this peak required a manual operation, with visual analysis or additional software in a second step after the routine identification process, and therefore impeding a real-time detection of KPC strains (Lau et al., 2014; Gaibani et al., 2016; Youn et al., 2016).

In this study, we present a full MALDI-based approach to an instant detection of KPC-producing *K. pneumoniae* strains simultaneously during the standard routine species identification process. KPC-producers were indirectly identified by the automated detection of a specific peak related to a *bla*_KPC_-carrying pKpQIL plasmid, using an algorithm that was integrated into the MALDI Biotyper software. A MALDI-TOF based imipenem hydrolysis assay was further used to confirm KPC enzyme activity.

This novel approach was applied to well characterized strains, to a large set of clinical routine strains, and finally directly to routine positive blood cultures.

## Materials and Methods

### Optimization of the Algorithm for Automated KPC Detection With PCR Confirmed Strains

**FIGURE 1:**
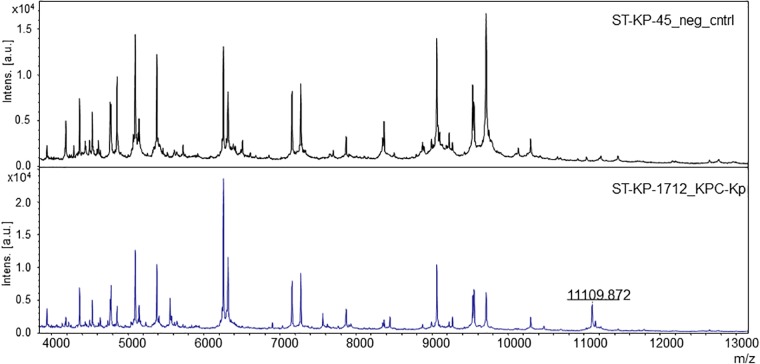
The pKpQIL plasmid-related peak in the MALDI mass spectra of *K. pneumoniae.* The upper spectrum shows a negative control, without the specific peak. The lower spectrum shows a KPC-producing strain exhibiting the specific peak.

**FIGURE 2:**
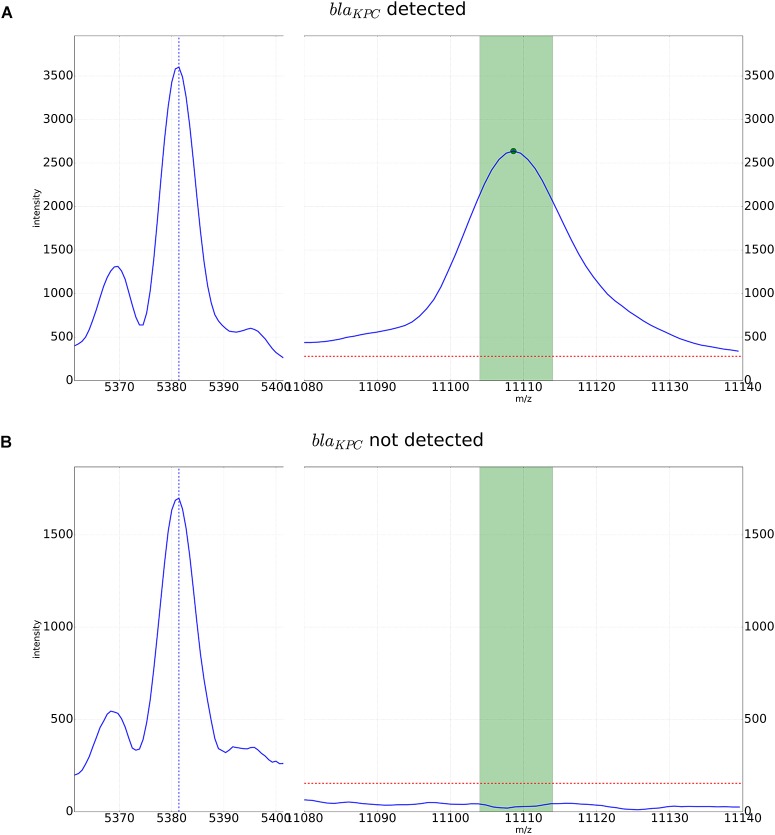
Detection of the KPC-related peak by the automated algorithm. An internal calibration peak specific to *K. pneumoniae* is recognized and used for higher precision in peak detection in the window of m/z 11,109 +/-5. The dotted red line corresponds to a multiple of the average noise in the spectrum. It is used as a threshold for the intensity in peak detection. **(A)** KPC-positive *K. pneumoniae* strain with peak for the pKpQIL plasmid. **(B)** KPC-negative strain. In the detection window only noise below the detection threshold can be observed.

For automated detection of the pKpQIL related peak described by Lau et al. (2014), a software algorithm was developed. The peak detection was based on a manual analysis of spectra analyzed with the flexAnalysis software version 3.4 (Bruker Daltonik, Bremen, Germany) – Figure 1. Spectra were normalized and smoothed with standard settings. For precise detection the spectra were screened for potential peaks for an internal recalibration as described in Pranada et al. (2017). After internal recalibration, intensities 3 times higher than the surrounding noise were then counted as peaks. If such potential peak was detected in a window ± 5 m/z around the previously described mass of 11,109 m/z, the algorithm yielded the detection of the KPC related peak. Examples of these automated detections are shown in Figure 2.

*N* = 266 spectra of *K. pneumoniae* previously characterized by PCR (Hyplex^®^ SuperBug ID assay – Amplex Biosystems GmbH, Gießen, Germany), were used to test the algorithm. Among them, *n* = 152 were KPC-producers, and *n* = 114 were negative for the *bla*_KPC_ gene.

To assess the technical sensitivity of the automatic detection, all the spectra were also analyzed by visual inspection using the FlexAnalysis software (Bruker Daltonik GmbH) to seek for the presence of the KPC-specific peak.

### Evaluation of the Automated KPC Detection With a Large Collection of Strains

The optimized algorithm was integrated into the commercial MALDI Biotyper RUO software, and the method was evaluated on a large set of clinical strains from Italy (in the Microbiology Unit of the University Hospital of Bologna Policlinico Sant’Orsola-Malpighi), as a country with a high prevalence of KPC-bearing strains, and from Germany (in the Department of Microbiology of the MVZ Dr. Eberhard & Partner Dortmund, Dortmund, Germany) as a low prevalence country.

A total of *n* = 6209 MALDI-TOF mass spectra of clinical and surveillance isolates of *K. pneumoniae* collected in a time frame between 2009 and 2017 were analyzed. The spectra were retrieved from the database of stored runs from routine identification in Dortmund. In Bologna, spectra for strains isolated between 2010 and September 2016 were recorded retrospectively from the collection of frozen strains as well as prospectively from October 2016 to December 2017.

Bacterial strains were cultured for 24–48 h on Tryptose Soy Agar with sheep blood in Bologna, and on Columbia Agar with sheep blood in Dortmund. Routine susceptibility testing was performed using VITEK2 XL systems (bioMérieux, Marcy l’Étoile, France).

All isolates with MICs greater than the EUCAST epidemiological cut-off for at least one of the carbapenems (ertapenem, meropenem, imipenem) underwent either detection of carbapenemase-encoding genes (in Dortmund by an in-house PCR) or confirmation of carbapenemase production, (in Bologna by disk diffusion synergy test with inhibitors (KPC+MBL Confirm ID Pack, ROSCO Diagnostika, Taastrup, Denmark), immunochromatographic assay (OXA-48 *K-*Set, CorisBioConcept, Gembloux, Belgium), and/or PCR (Hyplex^®^ SuperBug ID assay –Amplex Biosystems GmbH, Gießen, Germany, GeneXpert^®^ Carba-R –Cepheid, Sunnyvale, CA, United States).

Among these strains, at routine testing *n* = 2390 were KPC-producers (*n* = 2385 from Bologna, *n* = 5 from Dortmund), *n* = 179 MβL-producers (*n* = 119 from Bologna, *n* = 60 from Dortmund), *n* = 32 OXA-48 family producers (*n* = 19 from Bologna, *n* = 13 from Dortmund), *n* = 221 were negative for carbapenemase production but resistant to carbapenems (likely ESβL/AmpC producers associated with reduced membrane permeability – *n* = 207 from Bologna, *n* = 14 from Dortmund), and *n* = 3387 susceptible to carbapenems (β-lactamases producers or wild type – *n* = 105 from Bologna, *n* = 3282 from Dortmund) – Table 1.

**Table 1:** Overview of *K. pneumoniae* isolates from Italy and Germany included in this study.

	Italy	Germany	Total
	[n]	[n]	[n]
KPC	2386	4	2390
MβL	119	60	179
OXA-48	13	19	32
Carbapenem-resistant non-carbapenemase producers	207	14	221
Carbapenem-susceptible	105	3282	3387
Total	2830	3379	6209

### Evaluation of Stability and Reliability of KPC-Peak Detection

Further, the sensitivity of the automated KPC detection by MALDI-TOF MS was assessed with regard to the type of culture medium and the age of cultures: spectra of *n* = 34 KPC-producing strains cultured on Sheep Blood Agar, CHR-KPC Agar, and Mueller-Hinton-Agar (MEUS, Piove di Sacco, Italy) were acquired after 24, 48, and 72 h of incubation and then compared to each other.

Since the intensity of the KPC related peak in some cases is low, we also investigated whether the sensitivity of the automated detection could be enhanced measuring the samples in duplicates. For all samples, spectra were recorded from two spots.

In case of discrepancies between results of the novel MALDI approach and results of routine methods, the samples underwent molecular investigation by GeneXpert^®^ Carba-R (Cepheid, Sunnyvale, CA, United States).

### Application Directly to Positive Blood Cultures

The automated KPC detection was applied to 204 consecutive blood cultures positive for *K. pneumoniae* in routine diagnostics in Bologna. Among those, *n* = 90 were KPC-producers, *n* = 12 MβL-producers, *n* = 1 was NDM+OXA-48 producer, while *n* = 101 were susceptible to carbapenems (MIC of carbapenems lower than epidemiological cut-off). KPC-producers were identified by KPC *K-*Set, (CorisBioConcept, Gembloux, Belgium), MβL-producers were identified by KPC+MBL Confirm ID Pack, ROSCO Diagnostika, Taastrup, Denmark), while the strain NDM+OXA-48 was characterized by KPC+MBL Confirm ID Pack, ROSCO Diagnostika, Taastrup, Denmark and GeneXpert^®^ Carba-R (Cepheid, Sunnyvale, CA, United States).

The blood specimens were collected in BD BACTEC^TM^ blood culture media (Plus Aerobic/F, Plus Anerobic/F, Lytic/10 Anaerobic/F and Peds Plus^TM^/F), and incubated in a BACTEC^TM^ FX blood culture system (Becton, Dickinson and Company, Sparks, MD 21152, Benex Limited Shannon, County Clare, Ireland). After the bottles were flagged as positive, the routine workflow at the time of the study provided the species identification by MALDI-TOF MS after a subculturing step on chocolate agar of the enriched pellet obtained from 8 ml of blood culture.

For this study, the blood culture flasks were directly processed with the Sepsityper kit according to instructions of the manufacturer (Bruker Daltonik GmbH, Bremen, Germany) and the extracted bacterial pellet was used to acquire the MALDI spectra.

The KPC subtyping was performed by the MALDI Biotyper software simultaneously with the species identification.

The same pellet was further used to perform STAR-Carba assay (Bruker Daltonik GmbH) for carbapenemase confirmation.

### Confirmation of Carbapenemase Activity by STAR-Carba Assay

A subset of *n* = 243 randomly chosen (a representative proportion of strains for each year, selected at the discretion of the operator by manually choosing non-juxtaposed strains directly from the strains collection) KPC-producing strains, and all positive blood cultures containing *K. pneumoniae* (*n* = 204) underwent the MALDI-TOF MS based STAR-Carba imipenem hydrolysis assay (Bruker Daltonik GmbH, Bremen, Germany) to verify the carbapenemase activity. The test was performed according to the manufacturer’s instructions. Briefly, 1 μl-loop of bacteria (samples and *E.coli* ATCC 25922 as negative control, and a PCR-confirmed KPC-producing *E. coli* as positive control) were resuspended in 50 μl of imipenem solution. After 30 min of incubation at 37°C under agitation, the bacteria were pelleted by centrifugation (2 min at 14000 rpm), and 1 μl of the supernatant was spotted in duplicates onto a MALDI target. Open air dried spots were overlaid with CHCA matrix containing an internal standard. The strains were detected as hydrolyzing or non-hydrolyzing by measuring the intensity of the peak corresponding to the intact form of imipenem (300 m/z), normalized on the intensities values of the imipenem peak measured in the negative and positive controls.

## Results

### Optimization of the Algorithm for Automated KPC Detection With PCR Confirmed Strains

Internal calibration of the acquired MALDI-TOF mass spectra and optimization of the newly developed algorithm finally allowed narrowing down the detection window for the peak specific of the *bla*_KPC_-carrying pKpQIL plasmid to a more specific range of m/z 11,109 ± 5 (from initially ± 15).

The novel algorithm detected the *bla*_KPC_-carrying pKpQIL plasmid related peak in 99/152 PCR confirmed strains (65.1%).

The visual inspection of these spectra confirmed the presence of the peak in the 99 strains classified as positive by the algorithm (sensitivity 100%), while the remaining 53 strains didn’t show the peak, suggesting that they likely harbor a different *bla*_KPC_-carrying plasmid.

The KPC peak was not detected by the algorithm in the *n* = 114 KPC-PCR negative *K. pneumoniae* isolates (specificity 100%).

### Evaluation of the Automated KPC Detection With a Large Collection of Strains

The pKpQIL plasmid-related peak was detected by Biotyper software in overall 2035 of the *n* = 2390 KPC-producing isolates (85.1%). It was detected in none of the strains resulted positive for the production of a class B or D carbapenemase (*n* = 179 MβL, *n* = 32 OXA-48-family), in none of the carbapenem-susceptible isolates (*n* = 3387), but in 5 out of the *n* = 221 strains resulted resistant to carbapenems but negative to routine testing for carbapenemase production. The positive predictive value and the negative predictive value resulted 100% and 91.5%, respectively.

In these 5 strains, the molecular test confirmed the presence of *bla*_KPC_.

**FIGURE 3:**
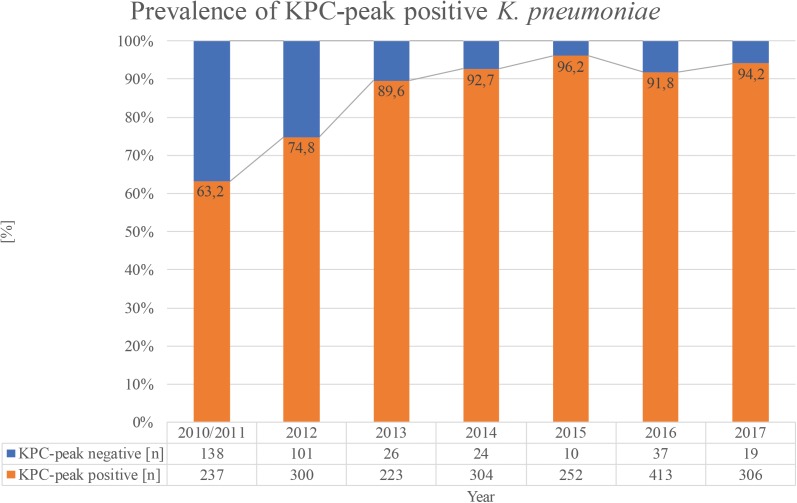
Trend for the years 2010–2017 of KPC-producing *K. pneumoniae* strains isolated in Bologna showing the pKpQIL plasmid-related peak.

With regard to the proportion of KPC-producing strains showing the peak among all KPC-producing isolates, a constantly increasing trend from 63.2% in the years 2010–2011 to 94.1% in 2017 could be observed (Figure 3).

### Evaluation of Stability and Reliability of KPC-Peak Detection

The evaluation of the KPC-detection with this huge set of spectra from clinical strains also comprised the investigation of culture conditions. No performance differences were observed in relation to culture medium used nor to time of incubation.

In 1798/2035 strains, and for 88.4% (1798/2035), the peak was detected in both spots. In 11.6% (237/2035) of the cases, the peak was detected only in one of the two spots.

### Application Directly to Positive Blood Cultures

To assess the further clinical potential, the automated KPC-detection was also applied directly on blood cultures. Among the spectra recorded from the bacterial pellet extracted by Sepsityper, the KPC-specific peak was detected in 83/90 (92.2%) of the KPC-producing strains, corresponding to 83/84 (98.8%) of the strains in which the peak was present.

The peak was detected in none of the non-KPC strains (100% specificity).

### Confirmation of Carbapenemase Activity by STAR-Carba Assay

STAR-Carba hydrolysis assay resulted positive for all the randomly chosen KPC-producing strains (*n* = 243).

It also resulted positive for all the KPC-producing strains from positive blood cultures (*n* = 90), as well as for all the other carbapenemase-producing strains (*n* = 12 MβL-producers, and *n* = 1 NDM+OXA-48-producer), and negative for the *n* = 101 carbapenem-susceptible strains from the blood cultures (sensitivity and specificity 100%).

## Discussion

Carbapenem-resistant enterobacteria are continuously spreading worldwide, and pose a serious threat to public health (Tzouvelekis et al., 2012; Nordmann, 2014). Carbapenemase-producing *Enterobacteriaceae* (CPE) represent an important cause of acquired infections in hospital settings, burdened by a high mortality rate (Vincent et al., 2009). Although the carbapenemases associated with *Enterobacterales* show a different prevalence in the different geographic areas, the KPC family has the most extensive global distribution, and it is the most significant (Munoz-Price et al., 2013; van Duin and Doi, 2017).

Rapid methods to detect KPC-producing strains are highly demanded, both for therapeutic and infection control purposes. Several methods are commercially available, relying on different principles, but overall they are either expensive, or slow, with time to report up to 24 h (Lutgring and Limbago, 2016; Tamma et al., 2017). Recently, a specific peak at 11,109 m/z, related to a pKpQIL plasmid carrying *bla*_KPC_ was discovered in the MALDI-TOF mass spectra of KPC-producing *Klebsiella pneumoniae* (Lau et al., 2014). Different approaches to seek for this peak have been described (Lau et al., 2014; Gaibani et al., 2016; Youn et al., 2016). Nevertheless all of them required a second step after routine species identification process like visual inspection or an additional software analysis.

In this study, we developed a fully MALDI-TOF MS based approach to detect KPC-producing *K. pneumoniae* strains. This novel approach relies on the indirect identification of *bla*_KPC_-carrying isolates by a now automated detection of the pKpQIL plasmid-related peak at 11,109 m/z during the standard routine identification process. The detection of the KPC-related peak is depicted in the MALDI Biotyper system as “presumptive KPC” in the subtyping column. Furthermore, we combined it with a subsequent verification of the carbapenemase activity by an imipenem hydrolysis assay, using the commercially available STAR-Carba kit, and according to the MALDI Biotyper software module (Bruker Daltonik, Bremen). The method was investigated on a large collection of strains from Italy and Germany – two countries with a very different epidemiology of CPE. The study aimed to evaluate this novel approach in a setting close to diagnostic routine, covering a broad period of time (2009–2017), in order to uncover pitfalls which can be missed by smaller dedicated study.

In addition, the integration of the algorithm for instant detection of the KPC-related peak into the MALDI Biotyper RUO software simplifies routine usage as no further special knowledge or additional tools are needed by the operator. The sensitivity of this automated method was found to be 85.1%. Our findings showed that it is mainly related to the prevalence of the KPC-producing strains harboring the pKpQIL plasmid among all the KPC-producing circulating strains. The visual inspection of the spectra included into the evaluation dataset of this study proved that the automated software detected the 11,109 m/z peak in all the strains in which it was present. For all strains in which the peak at 11,109 m/z was not detected by the automated subtyping algorithm, visual inspection of spectra confirmed this result. Moreover, a permanently increasing trend of the KPC-producing strains showing the pKpQIL specific peak was observed (from 60.2% in 2010–2011 to 95.6% in 2017), suggesting that the spread of KPC in Italy seems to be related to the expansion of this specific clone over the years. Although only 2/4 (50%) of the German KPC-producing strains showed the 11,109 m/z peak, this number is too low to draw conclusions about the prevalence of KPC in German strains. The method showed excellent specificity and positive predictive value (100%), as the software correctly classified all non-KPC carbapenemase-producers (*n* = 179 MβL and *n* = 32 OXA-48 family), as well as all carbapenem-susceptible isolates as KPC-peak negative. Among the strains resistant to carbapenems but negative for carbapenemase production in routine testing, 216/221 were classified as KPC-peak negative. In *n* = 5 strains the peak was detected, and for all of them the molecular testing confirmed the presence of *bla*_KPC_. Hence, interestingly, the method in study proved to be able to deliver an earlier detection of KPC carbapenemase in comparison with the other phenotypic methods applied. No differences were observed in the performance of the automated detection using different culture media (blood agar and chromogenic selective and non-selective media), or different incubation times (24–48–72 h), while the use of the spotting in duplicates enabled to achieve a better sensitivity in comparison to single spotting (85.1% vs. 80.2%). The failed detection of the peak is likely due to reasons linked to the sample preparation (operator technical handling, mucousity of the bacterial colonies). Therefore, spotting in duplicate might be advisable for increased robustness and sensitivity as minor biological or technical variations could be compensated.

Since the pKpQIL plasmid-related peak at 11,109 m/z is very well detectable in spectra of extractions, our study additionally investigated the performance of the method on positive blood cultures. Compared to colony material, the sensitivity of the peak detection on bacterial pellets extracted directly from positive blood culture using the Sepsityper protocol was 98.8% with an excellent specificity of 100%. This again underlines the value for clinical routine.

STAR-Carba imipenem hydrolysis assay was used with a subset of strains for confirmation of the carbapenemase activity, both in peak-positive and –negative KPC-producing isolates, and negative controls. It showed 100% sensitivity and specificity, from colonies as well as from the bacterial pellet extracted from positive blood cultures. In the approach we present here, this assay could play a fundamental role to detect carbapenemase activity, complementary to, and strengthening KPC peak detection. It can be used to detect carbapenemase activity in KPC-producing strains that don’t exhibit the presence of the KPC-related peak at 11,109 m/z, and in all the other carbapenemases of other classes. Further, it can be used as a functional verification of the enzymatic activity predicted by the presence of the KPC-marker detected by subtyping.

Our study shows that the novel MALDI-based approach enabled a reliable and robust detection of KPC-producing *K. pneumoniae* isolates, using the same MALDI Biotyper platform as for classical bacterial species identification, with a turnaround time from 10-15 min to 1.5 h. This method, applicable to colony material as well as directly on positive blood cultures, is unique in performing real time detection of an antibiotic resistance marker in parallel to species identification. It might represent a very useful tool for an early warning for KPC-producing strains, helping to significantly accelerate the proper initiation or change of therapeutic and infection control measures in the future. In comparison to previously described methods based on the 11,109 m/z peak detection, the approach we developed presents several advantages. First, the peak detection here is implemented into a commercially available software, and totally automated. Thereby, it doesn’t require any operator specific skills regarding spectra processing and analysis. The STAR Carba assay is easy to use, and requires a minimum handling time. Moreover, the interpretation of results is also automated, and hence free from any operator-depending factors, and results are automatically stored and available for eventual future investigation. Thus, this approach can fit with any setting, including high throughput routine laboratories.

The limitation of the presented approach is that the KPC detection by subtyping depends on the association of the peak to *bla*_KPC_ gene. Partridge (2014) pointed out that BLAST searches indicated that the underlying p019 protein has only been found in plasmids carrying *bla*_KPC_ but an independent genetic movement of the p019 gene might be possible in the future and then impair this indirect detection method. A further limitation is that the sensitivity of the subtyping approach depends on the regional epidemiology of KPC-producing strains, i.e., on the prevalence of the KPC-producing clones harboring the pKpQIL plasmid. Nevertheless, the excellent specificity, and the seamless integration into the commercial software already used for bacterial species identification, might make the implementation of this approach in routine valuable, regardless the current epidemiological context.

Further, as KPC-producing strains have started appearing also among other genera and species of enterobacteria, a future expansion of this approach to other species might be possible.

## Data Availability

The authors state that datasets are available on request. The raw data supporting the conclusion of this manuscript, will be made available by the authors, without undue reservation, to any qualified researcher.

## Author Contributions

MC, MK, and ABP designed the study. MC, MK, and ABP carried out the study. MB and JG worked on the computer algorithms and statistical analysis together with MC and ABP. MC, MK, ABP, and SA wrote the manuscript.

## Conflict of Interest Statement

MK and JG are employees of Bruker Daltonik GmbH, the manufacturer of the MALDI Biotyper system. The remaining authors declare that the research was conducted in the absence of any commercial or financial relationships that could be construed as a potential conflict of interest.
